# Ultrasound-Assisted Dispersive Solid-Phase Filter Extraction Coupled with Green Supercritical Fluid Chromatography Methodology for Simultaneous Determination of Hindered Phenolic Antioxidant Migration from Food Contact Materials

**DOI:** 10.3390/foods14132301

**Published:** 2025-06-28

**Authors:** Shaojie Pan, Chaoyan Lou, Xiaolin Yu, Kaidi Zhang, Kai Zhang, Lei Jiang, Yan Zhu

**Affiliations:** 1 College of Quality and Standardization, China Jiliang University, Hangzhou 310018, China; 18868730104@163.com (S.P.); unffgg@163.com (X.Y.); 15098639069@163.com (K.Z.); 2 Ningbo Key Laboratory of Agricultural Germplasm Resources Mining and Environmental Regulation, College of Science and Technology, Ningbo University, Ningbo 315300, China; zhangkai1@nbu.edu.cn; 3 Zhejiang Institute of Quality Sciences, Hangzhou 310018, China; jxst985792@163.com; 4 Department of Chemistry, Zhejiang University, Hangzhou 310028, China; yan@zju.edu.cn

**Keywords:** food contact materials, food safety, green chemistry, hindered phenolic antioxidants, migration assessment, supercritical fluid chromatography, ultrasound-assisted dispersive solid-phase filter extraction

## Abstract

The migration of hindered phenolic antioxidants from food contact materials (FCMs) into foodstuffs poses health risks due to endocrine disruption and organ toxicity. Hence, the development of a high-efficiency analytical method for hindered phenolic antioxidants is of great importance for food safety. This study developed a novel ultrasound-assisted dispersive solid-phase filter extraction (d-SPFE) coupled with green supercritical fluid chromatography (SFC) method for the simultaneous determination of six representative hindered phenolic antioxidants. Under optimized conditions, the method achieved high extraction efficiency, with the complete separation of all analytes within 10 min. A wide linearity range (0.02–2.0 μg/mL) was achieved, with coefficients of determination all greater than 0.9996. The limits of detection (LOD, S/N = 3) and limits of quantification (LOQ, S/N = 10) were 2.4–3.6 ng/mL and 8–12 ng/mL, respectively. Validation tests demonstrated precise spiked recoveries (89.4–101.6%), with intra-day and inter-day relative standard deviations (RSDs) all less than 10%. The d-SPFE-SFC synergy significantly outperforms conventional techniques in terms of analysis speed and eco-efficiency. Successful application to food simulants confirms its reliability in monitoring hindered phenolic antioxidant migration from FCMs. This green and rapid methodology will enable the direct assessment of migration risks.

## 1. Introduction

In the food industry, antioxidants are a class of chemical additives that are widely used in the manufacturing of food contact materials (FCMs) to enhance their oxidative stability and extend their lifespan [1,2]. Hindered phenolic antioxidants are some of the most frequently used synthetic antioxidants due to their good oxidation resistance and high cost-effectiveness [3]. However, these antioxidants are not covalently bonded with food contact materials during manufacturing processes, creating significant migration risks in food products. This potential migration behavior has raised substantial public concerns regarding human exposure through dietary intake and potential bioaccumulation in the food chain [4,5]. The long-term intake of hindered phenolic antioxidants can cause adverse effects on human health, since these antioxidants are carcinogenic, mutagenic, and endocrine-disrupting [6,7]. As a consequence, the specific migration limits (SMLs) of antioxidants from FCMs to foodstuffs or food simulants are strictly regulated in various countries and regions (e.g., FDA FCM/FCN/Food Contact Notification and EU Regulation No. 10/2011 on plastic materials and articles intended to come into contact with foodstuffs) [2]. For instance, China mandates specific migration limits on hindered phenolic antioxidants as follows: 30 mg/kg for butylated hydroxyanisole (BHA), 6 mg/kg for antioxidant 1790 (AO 1790), and 1.5 mg/kg for both antioxidant 2246 (AO 2246) and antioxidant 425 (AO 425) [8]. Notably, the development of environmentally friendly analytical methods with high sensitivity and efficacy is critical in characterizing antioxidant migration behavior from FCMs to foodstuffs.

However, monitoring hindered phenolic antioxidants in foodstuffs or food simulants remains challenging due to matrix interference and trace-level migration quantification from FCMs. Consequently, highly efficient sample pretreatment is essential to accurately analyze these antioxidants at low concentrations. Among various sample pretreatment strategies, solid-phase extraction (SPE) [9,10,11] is extensively used for matrix cleanup and analyte concentration. Several novel SPE techniques have been reported for the preconcentration and cleanup of antioxidants prior to analysis, including dispersive solid-phase extraction (d-SPE) [12,13], magnetic solid-phase extraction (MSPE) [14,15], molecularly imprinted solid-phase extraction (MISPE) [16], on-line solid-phase extraction (on-line SPE) [17,18,19], and QuEChERS [20,21]. Despite their utility, these methods share inherent limitations: laborious procedures, significant sorbent consumption, and large volumes of organic solvents. To overcome these constraints and provide a more efficient alternative, an innovative extended SPE method called dispersive solid-phase filter extraction (d-SPFE) is introduced in this study. Dispersive solid-phase filter extraction [22,23] is a hybrid approach, synergistically integrating the rapid filtration mechanism with the dispersion-enhanced kinetics of d-SPE. The adsorption and elution processes of d-SPFE are operated in a syringe, with rapid separation achieved between the solution and sorbent via the filter head, eliminating the need for centrifugation. Compared with commercially available SPE columns, d-SPFE is an inexpensive and miniaturized procedure that requires fewer organic solvents and sorbents.

Moreover, a comprehensive green analytical strategy for hindered phenolic antioxidants not only requires an efficient sample pretreatment technique but also an eco-friendly instrumental method. At present, high-performance liquid chromatography (HPLC) [17,24], liquid chromatography coupled with mass spectrometry (LC-MS/MS) [25,26], ultra-high-performance liquid chromatography coupled with mass spectrometry (UPLC-MS/MS) [4,14], and colorimetric sensor arrays [27] are used to detect hindered phenolic antioxidants. Although these instrumental approaches can meet sensitivity and reproducibility requirements, they require large volumes of organic solvents and long analysis times. To address these limitations, a supercritical fluid chromatography (SFC) method is introduced for the simultaneous determination of six hindered phenolic antioxidants. SFC is an emerging chromatography method using supercritical fluids as the mobile phase, primarily carbon dioxide [28,29]. The low viscosity and high diffusivity of supercritical fluids facilitate faster mass transfer between the mobile and stationary phases compared with conventional liquid chromatography, significantly reducing the running times without compromising the resolution [30]. Additionally, due to the adjustability and versatility of the mobile phase, SFC is beneficial in separating a broad range of compounds, from polar pharmaceuticals to non-polar lipids. Moreover, unlike traditional methods that require large volumes of toxic organic solvents, SFC’s reliance on carbon dioxide makes it an environmentally sustainable alternative.

In the present study, an ultrasound-assisted dispersive solid-phase filter extraction strategy combined with a supercritical fluid chromatography methodology is proposed for the green and rapid measurement of six frequently used hindered phenolic antioxidants that can migrate from food contact materials (BHA, antioxidant 33, antioxidant 246, antioxidant 2246, antioxidant 425, and antioxidant 1790). Both the d-SPFE operating parameters and the SFC conditions were investigated to achieve optimal performance for qualitative and quantitative analysis. Subsequently, the proposed method was applied for the migration study of hindered phenolic antioxidants in food contact materials. The results demonstrate that the proposed approach features several advantages, including small amounts of sorbents, less organic solvent consumption, reduced operational time, and portable miniaturization, fulfilling the principles of green chemistry and high-throughput analysis requirements. Moreover, from a food safety perspective, this green and convenient methodology’s application scope could be expanded to monitor other additives that migrate from food contact materials.

## 2. Materials and Methods

### 2.1. Chemicals and Materials

**Figure 1:**
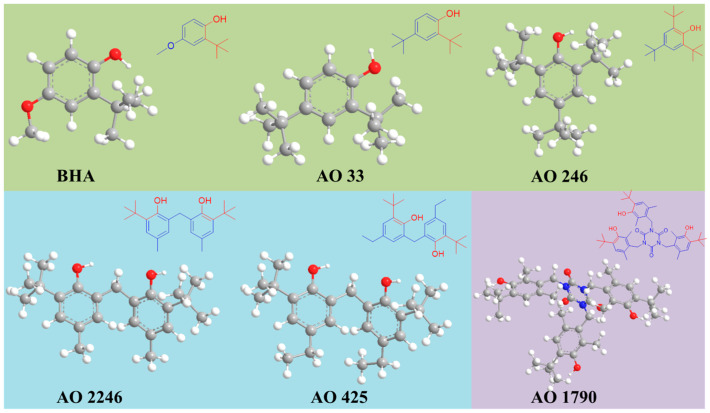
The stereochemical structures of the investigated hindered phenolic antioxidants. These antioxidants are classified into three subclasses: hindered monophenolic antioxidants (green background), hindered bisphenolic antioxidants (blue background), and hindered polyphenolic antioxidants (purple background).

Six hindered phenolic antioxidants, namely, butylated hydroxyanisole (BHA), 2,4-di-tert-butylphenol (AO 33), 2,4,6-tri-tert-butylphenol (AO 246), 2,2′-methylenebis(6-tert-butyl-4-methylphenol) (AO 2246), 2,2′-methylenebis(4-ethyl-6-tert-butylphenol) (AO 425), and Tris(4-tert-butyl-3-hydroxy-2,6-dimethylbenzyl) isocyanurate (AO 1790), were purchased from different companies. BHA, AO 33, AO 246, and AO 425 were purchased from the Aladdin Biochemical Technology Corporation (Shanghai, China), while AO 2246 and AO 1790 were supplied by the Macklin Biochemical Technology Corporation (Shanghai, China) and Bide Pharmaceutical Technology Corporation (Shanghai, China), respectively. Molecular formulas, chemical structures, and additional information for these hindered phenolic antioxidants are presented in Table 1 and Figure 1. The organic reagents used in the experiments, including methanol, acetonitrile, and ethanol, were of HPLC grade and provided by the Tedia Company (Fairfield, CT, USA). Ultrapure carbon dioxide (purity ≥ 99.999%) was obtained from the Hangzhou Jingong Special Gas Corporation (Hangzhou, China). Deionized water was produced by the Millipore Simplicity System (Burlington, MA, USA).

Moreover, homemade sorbents based on polyethylvinylbenzene/divinylbenzene (EVB/DVB) microspheres were prepared in the laboratory according to our previously reported two-step swelling and polymerization method [31]. Commercial sorbents including N-propylethylenediamine (PSA, 40–60 µm) and graphitized carbon black (GCB, 38–124 µm) were purchased from Agela Technologies (Tianjin, China). Injection syringes and hydrophobic membrane filters (pore size 0.22 μm) were purchased from Thermo Fisher Scientific (Waltham, MA, USA). The food contact materials investigated in the experiments were commercial plastic containers, which were purchased from the suppliers. All these samples were handled with laboratory glassware to avoid potential contamination from plastic wares. Moreover, all involved experimental consumables, including filters and containers, were washed thoroughly with deionized water before use to remove potential interference.

### 2.2. Instruments and Analytical Conditions

Six hindered phenolic antioxidants were simultaneously determined on a Nexera UC Supercritical Fluid Chromatography System (Shimadzu, Kyoto, Japan).This system was equipped with an auto-sampler unit, a supercritical fluid delivery unit, a liquid delivery unit, a column oven, and a backpressure control unit. The SFC system was configured with a UV detector and controlled using a LabSolutions workstation (Ver 5.8). Furthermore, a numerical control ultrasonic cleaner (KQ-500DE) (Kunshan Ultrasound Instrument, Shanghai, China) was utilized for sample ultrasonic treatment.

The separation and measurement of hindered phenolic antioxidants was achieved under the following chromatographic conditions: an Acclaim 120 C18 (5 μm, 4.6 mm × 250 mm) column was utilized as the stationary phase at a maintained temperature of 38 °C. In the meantime, 10 MPa of backpressure was forced through the system to maintain the supercritical status. The injection volume was 5 μL and the wavelength of UV detection was set at 220 nm. As for the mobile phase, a gradient elution program was preferred to guarantee adequate separation. The detailed gradient elution program is depicted in Table S1.

### 2.3. Preparation of Standard Solutions and Food Simulants

The antioxidant content in food simulants was quantified using the external standard method. A standard stock solution was precisely prepared using the weighing method and stored in a dark refrigerator at 4 °C. A series of standard working solutions at the required concentrations were obtained via the stepwise dilution of the standard stock solution.

Food simulants are laboratory substances used to mimic the behavior of real foods in migration studies. In accordance with the standards of the European Union (EN 13130-1:2004 [32]) and China (GB/T 23296.1-2009 [33]), three different kinds of food simulants were prepared to investigate the specific migration levels of antioxidants from food contact materials. Specifically, pure water (Simulant A) was used to simulate real aqueous foods, a 3% acetic acid aqueous solution (Simulant B) was used to substitute real acidic foods, and a 10% ethanol aqueous solution (Simulant C) was used to substitute alcoholic foods.

### 2.4. Simulated Migration Experiment

The specific migration tests for hindered phenolic antioxidants were conducted according to the guidelines from EN 13130-1:2004 [32] and GB/T 23296.1-2009 [33]. Six plastic packaging material samples were collected for further migration tests. Each sample of 0.6 dm^2^ was cut into small pieces (20 mm × 20 mm) and immersed into 100 mL of food simulant with double-sided contact, maintained at 40 °C for 10 durative days. After completing the treatment, the FCMs were strained off from the solution using a nylon filtration membrane (pore size: 0.22 μm), and the migrated simulants were further prepared via dispersive solid-phase filtered extraction.

### 2.5. Operation of Ultrasound-Assisted Dispersive Solid-Phase Filter Extraction

**Figure 2:**
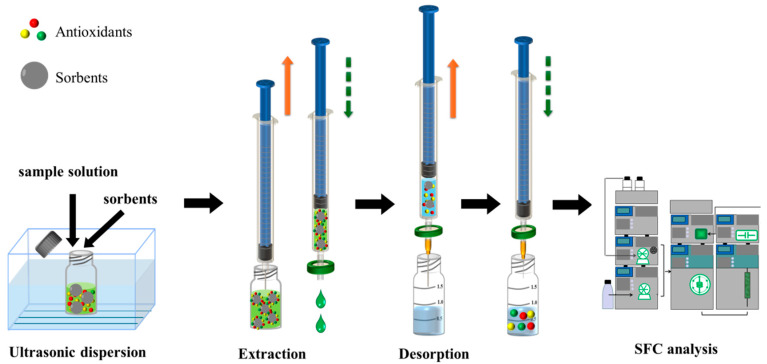
Schematic of ultrasonic-assisted dispersive solid-phase filter extraction with supercritical fluid chromatography.

The d-SPFE procedure is depicted in Figure 2. In total, 8.0 mg of EVB/DVB microspheres was accurately weighed and added into a glass vial filled with 5.0 mL of sample solution. The mixed solution was ultrasonicated at room temperature for 5 min to achieve full dispersion and adsorption. The mixture was loaded into a syringe and attached to a disposable organic filter. The plunger was then pushed constantly to separate the sorbents and solvent. Subsequently, a needle was attached to the syringe and 1.0 mL of acetonitrile was drawn into the syringe to desorb the hindered phenolic antioxidants. The plunger was pushed and pulled for three cycles to adequately elute the target analytes from the sorbents. Finally, the collected elution solvent was dried and reconstituted in 0.5 mL for further SFC analysis.

## 3. Results and Discussion

### 3.1. Investigation of d-SPFE Operating Parameters

To achieve optimal extraction efficiency for these hindered phenolic antioxidants, we investigated the effects of different parameters on their performance. The conditions were optimized, including the selection of sorbent types, sorbent dosage, adsorption time, desorption solvent type, and desorption parameters. All experiments were conducted in triplicate.

#### 3.1.1. Selection of Sorbent Types

**Figure 3:**
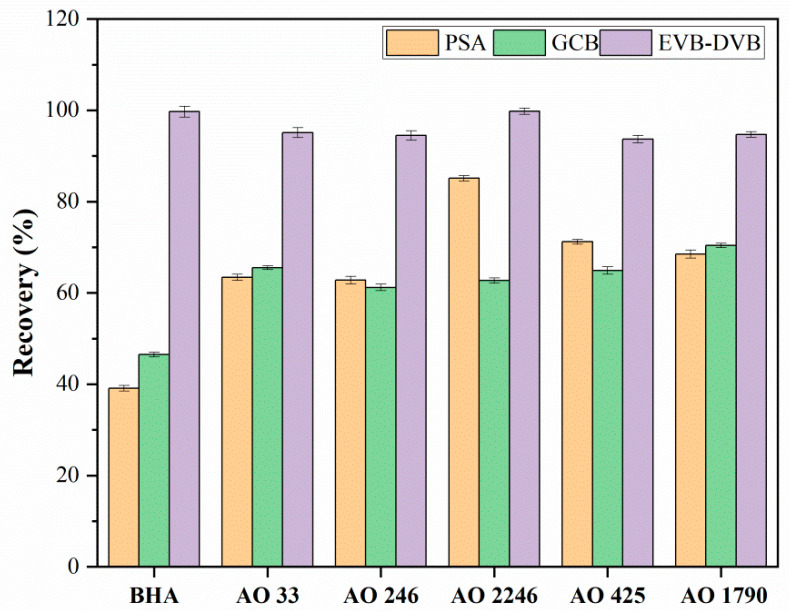
Extraction efficiency of different sorbents (n = 3). Recovery was determined as follows: (C_measured_/C_spiked_) × 100%, where C_measured_ denotes the measured value and C_spiked_ denotes the theoretical spiked concentration.

In dispersive solid-phase filter extraction, the most crucial factor for extraction efficiency is the sorbent. The effects of both commercial sorbents (PSA, GCB) and the laboratory-made EVB/DVB sorbent were tested and compared. Each sorbent (8.0 mg) was weighed and added to glass vials containing 5.0 mL of a 0.5 μg/mL spiked solution to carry out the d-SPFE procedures. The results in Figure 3 indicate that, when EVB/DVB was used as the sorbent, the highest recovery for the six compounds was achieved. Several factors could account for this result. Firstly, hindered phenolic antioxidants are organic aromatic compounds with low polarity; hence, EVB/DVB, with rich benzene rings in its skeleton frame, was preferred due to the hydrophobic interactions and enhanced π–π interactions between them. Moreover, the specific surface area of EVB/DVB (210 m^2^/g) significantly exceeded that of GCB (100 m^2^/g), providing sufficient active binding sites for the antioxidants. Consequently, EVB/DVB was ultimately selected as the extraction sorbent for subsequent experiments.

#### 3.1.2. Study of Absorption Conditions

**Figure 4:**
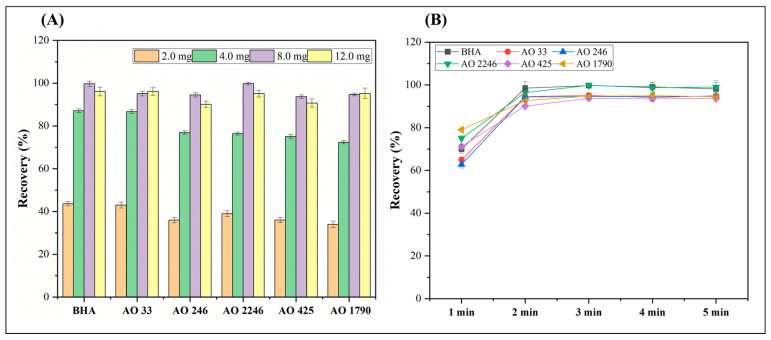
Effects of different parameters on absorption efficiency (n = 3). (**A**) Effect of sorbent dosage and (**B**) effect of absorption time.

Both the dosage of sorbent and absorption time were investigated to completely absorb the hindered phenolic antioxidants. Four EVB/DVB microsphere amounts (2.0, 4.0, 8.0, and 12.0 mg) were chosen to investigate the extraction efficiency. According to the recovery results in Figure 4A, the extraction recovery of the antioxidants increased when the dosage of EVB/DVB increased from 2.0 mg to 8.0 mg. Furthermore, when the quantity of EVB/DVB continuously expanded from 8.0 mg to 12.0 mg, the adsorption efficiency did not significantly increase, indicating that 8.0 mg of EVB/DVB was sufficient for the optimal extraction of the hindered phenolic antioxidants. An excessive sorbent amount of 12.0 mg was not considered in this work since this dosage not only results in resource overconsumption but can also cause filter clogging. Thus, 8.0 mg of EVB/DVB was selected as the optimal dosage.

The adsorption time represented the equilibrium time for hindered phenolic antioxidants accumulating on EVB/DVB. In this experiment, the adsorption time was systematically studied from 1 to 5 min. As illustrated in Figure 4B, an absorption equilibrium was achieved within only 3 min and no significant improvements were observed beyond this threshold. Such fast mass transfer is beneficial for extraction efficiency, so 3 min was selected as the preferred adsorption time.

#### 3.1.3. Study of Desorption Conditions

**Figure 5:**
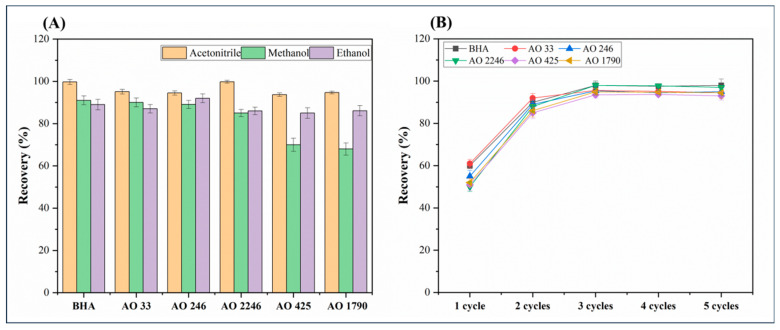
Effects of different parameters on desorption efficiency (n = 3). (**A**) Selection of desorption solvent and (**B**) study of desorption time.

The desorption conditions were also investigated, including the selection of solvent types and desorption parameters. Three regularly used desorption solvents, namely acetonitrile, methanol, and ethanol, were taken into consideration to elute antioxidants from EVB/DVB sorbents. As shown in Figure 5A, acetonitrile achieved superior recovery rates for all six antioxidants compared with the other two solvents. Consequently, acetonitrile was selected as the optimal desorption solvent.

Furthermore, the number of required cycles to push and pull the syringe plunger was also evaluated. The desorption frequency was tested in the scale of one to five cycles. As shown in Figure 5B, when the push and pull operation increased to three cycles, it was sufficient to wash off all six antioxidants, and prolonging the operation did not significantly affect the recovery rate. Therefore, three cycles of pushing and pulling the syringe plunger were enough for the desorption operation.

### 3.2. Optimization of Supercritical Fluid Chromatography Conditions

Supercritical fluid chromatography offers a unique combination of advantages that stem from its use of supercritical fluids as the mobile phase, primarily carbon dioxide. Unlike traditional methods that require large volumes of toxic organic solvents, SFC minimizes hazardous waste generation and aligns with green chemistry principles. To achieve high efficiency and the rapid analysis of hindered phenolic antioxidants, SFC conditions including the modifier type, percentage of modifier, backpressure, and temperature were studied in this part. The hindered phenolic antioxidants were aromatic compounds with weak polarity. Given the weak polarity, a C18 column was preferentially adopted as the stationary phase to separate these antioxidants.

The mobile phase is another critical parameter that determines the separation of hindered phenolic antioxidants. As presented in Figure 1, hindered phenolic antioxidants are structurally similar compounds characterized by a phenolic group together with tertiary butyl hindrance. Although the low viscosity and high diffusivity of supercritical fluids facilitate faster mass transfer between the mobile and stationary phases compared with conventional liquid chromatography, significantly reducing the run times without compromising the resolution, it is non-negligible that the non-polar nature of pure supercritical CO_2_ constrains its ability to solvate and elute hindered phenolic antioxidants. To deal with this condition, polar organic solvents are suggested as modifiers to enhance the elution strength of the mobile phase. In this experiment, three different solvents, namely methanol, ethanol, and acetonitrile, were evaluated as modifiers. The results revealed methanol’s superior performance, delivering the optimal resolution, peak symmetry, and analysis time. Ethanol induced higher system pressures due to its increased viscosity, while acetonitrile failed to achieve baseline separation for AO 2246, AO 425, and AO1790. Based on these findings, methanol was selected as the mobile phase modifier.

**Figure 6:**
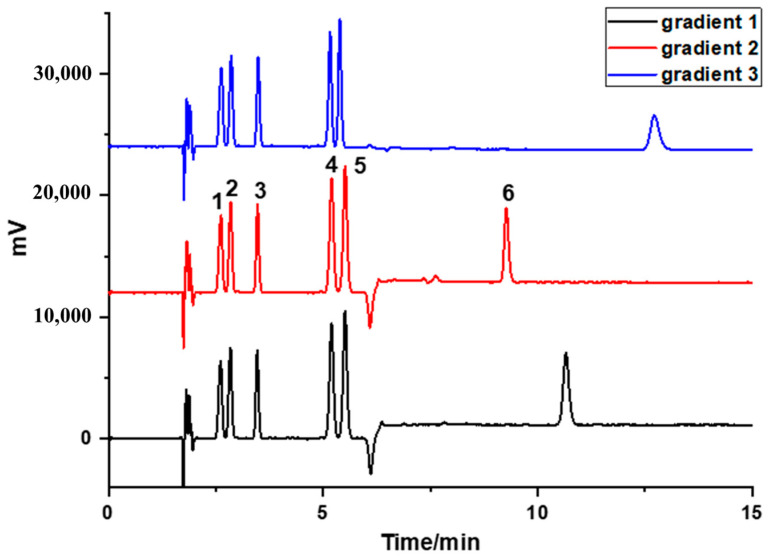
Study of gradient elution conditions. Column temperature: 38 °C; BPR: 10 MPa; Peak: (1) BHA, (2) AO 33, (3) AO 246, (4) AO 2246, (5) AO 425, and (6) AO 1790.

The proportion of methanol was further optimized. Due to the relatively low polarity of the target antioxidants, a small quantity of methanol was used to modulate the elution strength. Different percentages of methanol ranging from 3% to 10% were tested and compared. The elution order correlated with the number of hindered phenolic groups in the antioxidant structure. The retention was enhanced as the hindered phenolic groups increased. At 5% methanol, baseline separation was achieved for the three monophenolic antioxidants. However, this concentration proved insufficient for the adequate separation of bisphenolic antioxidants and polyphenolic antioxidants. On this basis, a gradient elution mode was used to ensure the effective separation of all six antioxidants. Three alternative gradient elution conditions (depicted in Table S2) were proposed and explored to achieve the optimum separation performance. As shown in Figure 6, although the hindered phenolic antioxidants could achieve baseline separation under all three gradient programs, gradient 2 provided the shortest analysis time and the narrowest, most symmetrical peaks.

In addition to the stationary phase and mobile phase, other supercritical parameters, including the backpressure and temperature, were also optimized. The backpressure is a unique parameter in SFC. Typically, the density of the supercritical fluid increases with the backpressure, contributing to enhanced solvation effects. In this study, the backpressure of 10 MPa was adopted to ensure the effect. The temperature was optimized by comprehensively considering the analysis time and separation resolution. After testing the temperature from 34 °C to 40 °C, 38 °C was chosen for subsequent experiments to meet the requirements of rapid analysis and a high resolution. Under these optimized conditions, the six antioxidants were isolated within 10 min.

### 3.3. Methodological Validation and Evaluation

The performance of the proposed method was validated by systematically evaluating the key analytical parameters. As shown in Table 2, a linear relationship between the analyte signal and concentration was observed in a range of 0.02–2.0 μg/mL, established through a seven-point calibration curve (0.02, 0.05, 0.1, 0.2, 0.5, 1.0, and 2.0 μg/mL). The coefficients of determination (R^2^) exceeded 0.9996 for all analytes. The limits of detection (LOD), defined as three folds of signal-to-noise (S/N = 3), were calculated as 2.4–3.6 ng/mL, while the limits of quantification (LOQ), defined as ten folds of signal-to-noise (S/N = 10), were calculated as 8–12 ng/mL.

The method’s precision and accuracy were also estimated through spiking recovery tests and repeatability studies. Spiking recovery experiments were conducted at low (20 ng/mL), medium (100 ng/mL), and high (500 ng/mL) concentration levels in simulants A, B, and C, respectively. Intra-day precision was assessed by analyzing five replicates on the same day, and inter-day precision was assessed over five consecutive days. The results in Table 3 demonstrate that the spiked recoveries of six hindered phenolic antioxidants were at a scale of 89.4% to 101.6%. Both the inter-day and intra-day RSDs were less than 10%. These results confirm the reliability and robustness of the proposed method for qualitative and quantitative analysis.

### 3.4. Method Application in Migration Analysis

The established method was applied to measure hindered phenolic antioxidants that migrate from food contact materials. Six commercially available plastic packaging samples were selected and prepared. The results in Table S3 show that two typical hindered phenolic antioxidants, named AO 246 and AO 2246, were most frequently detected in the samples, and other antioxidants, like BHA and AO 33, were not detected (below the detectable limit). The migration amounts of AO 246 and AO 2246 were at a scale of 0.471 to 1.397 mg/kg. Although the migration levels comply with the SMLs in the Chinese national standards and EU regulations, migration risks are still a significant concern regarding chronic consumer exposure. These findings underscore the critical importance of controlling food storage conditions and selecting appropriate packaging materials to mitigate migration risks. In this case, further investigation is required to elucidate the effects of the storage time and storage temperature on antioxidant migration behavior.

### 3.5. Preliminary Migration Study of Hindered Phenolic Antioxidants

**Figure 7:**
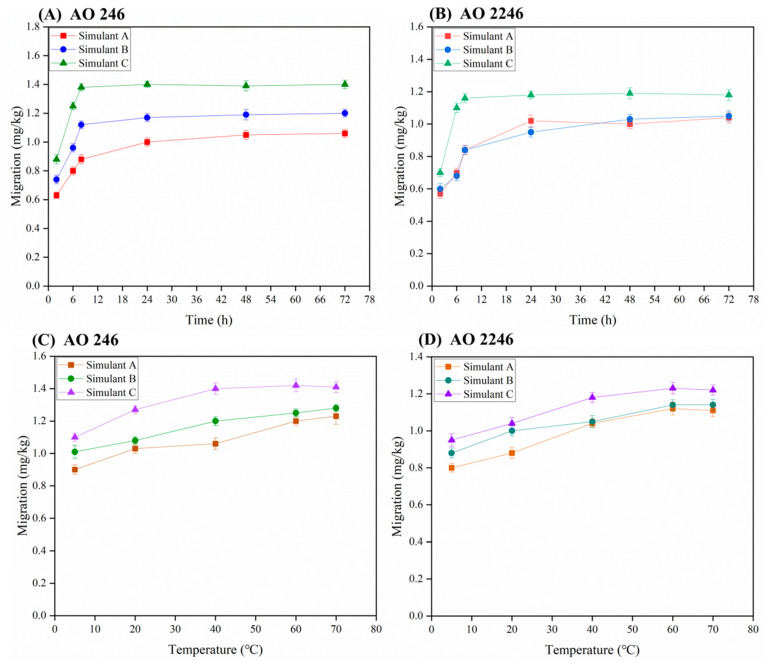
The effects of the storage time and temperature on migration. (**A**) Migration behavior of AO 246 over time, (**B**) migration behavior of AO 2246 over time, (**C**) migration behavior of AO 246 at different temperatures, and (**D**) migration behavior of AO 2246 at different temperatures.

Positive food packaging samples were selected to investigate the migration behavior of hindered phenolic antioxidants related to the storage time and temperature. The effect of the storage time on migration was investigated at 2, 6, 8, 24, 48, and 72 h. Figure 7A,B show the migration of AO 246 and AO 2246 over time. The migration of these two antioxidants initially increased and then stabilized over time. The rapid migration period occurs within initial 12 h, and a migration equilibrium is basically achieved within 24 h. The temperature is another significant factor that influences the migration behavior. Hence, simulants immersed in food contact materials were stored at 5 °C, 20 °C, 40 °C, 60 °C, and 70 °C, and the antioxidant content was determined using the d-SPFE-SFC method. Figure 7C,D indicate that the migration levels of these two hindered phenolic antioxidants increased with the temperature. Moreover, by comparing Simulant A (water), Simulant B (3% acetic acid aqueous solution), and Simulant C (10% ethanol aqueous solution), it could be found that the alcoholic matrix promoted the migration of these two antioxidants. In conclusion, it is suggested to pay attention to the storage conditions and food types during the selection of FCMs in the food industry.

### 3.6. Comparison with Reported Methods

The method proposed in this study was compared with previously reported methods in the literature. The key metrics are summarized in Table 4. Compared with other approaches, d-SPFE is a streamlined preparation method that requires minimal sorbent (8 mg) and achieves efficient extraction within 8 min. The extraction process is operated using a syringe, offering the advantages of miniaturization and operational simplicity. Complementing these advantages, the supercritical fluid chromatography protocol delivers rapid separation (10 min), avoids the large consumption of toxic organic solvents, and enhances the environmental sustainability. On the other hand, the sensitivity of this method can be further improved by combining it with mass spectrometric detection. In general, it is a rapid, easily operated, green, and inexpensive approach for the analysis of antioxidants in the food field.

## 4. Conclusions

The migration of hindered phenolic antioxidants from food contact materials (FCMs) into food poses significant health risks, necessitating efficient monitoring tools. This study established a green and robust methodology for the rapid determination of six hindered phenolic antioxidant migrants in food simulants by integrating dispersive solid-phase filter extraction (d-SPFE) with supercritical fluid chromatography (SFC). Rigorous validation confirmed the wide linearity (0.02–2.0 μg/mL, R^2^ ≥ 0.9996), good sensitivity (LODs 2.4–3.6 ng/mL), and high accuracy (89.4–101.6% recovery), with high precision (RSD < 10%) across acidic, ethanolic, and aqueous matrices. This approach was successfully applied to monitor antioxidant migration in commercial food contact materials. Preliminary studies demonstrated that the migration behavior of hindered phenolic antioxidants was dependent on the storage conditions and food matrix types. Compared with reported works, this proposed approach enables operational convenience, high-throughput analysis, and reduced organic solvent consumption, showing potential for the monitoring of other additives that migrate from food contact materials. Moreover, this method could be used to predict emerging contaminants by integrating it with frontier intelligent technology.

## Figures and Tables

**Table 1:** Hindered phenolic antioxidants studied in this work.

Hindered Phenolic Antioxidant	Chemical Compound	Abbreviation	CAS No.	Molecular Formula	Molecular Weight	Classification
BHA	Butylated hydroxyanisole	BHA	25013-16-5	C_11_H_16_O_2_	180.2	Monophenolic antioxidant
Antioxidant 33	2,4-Di-tert-butylphenol	AO 33	96-76-4	C_14_H_22_O	206.3	Monophenolic antioxidant
Antioxidant 246	2,4,6-Tri-tert-butylphenol	AO 246	732-26-3	C_18_H_30_O	262.4	Monophenolic antioxidant
Antioxidant 2246	2,2′-Methylenebis(6-tert-butyl-4-methylphenol)	AO 2246	119-47-1	C_23_H_32_O_2_	340.5	Bisphenolic antioxidant
Antioxidant 425	2,2′-Methylenebis(4-ethyl-6-tert-butylphenol)	AO 425	88-24-4	C_25_H_36_O_2_	368.6	Bisphenolic antioxidant
Antioxidant 1790	Tris(4-tert-butyl-3-hydroxy- 2,6-dimethylbenzyl) isocyanurate	AO 1790	40601-76-1	C_42_H_57_N_3_O_6_	699.9	Polyphenolic antioxidant

**Table 2:** Linear ranges, coefficients of determination, limits of detection, and limits of quantification for the six antioxidants in this study.

Antioxidant	Linear Range (μg/mL)	Slope ± SE * (×10^3^)	Intercept ± SE	R^2^	LOD (ng/mL)	LOQ (ng/mL)
BHA	0.02–2.0	32.75 ± 0.36	268.3 ± 5.2	0.9996	3.6	12
AO 33	0.02–2.0	34.65 ± 0.38	−526.6 ± 8.3	0.9998	3.5	12
AO 246	0.02–2.0	31.28 ± 0.35	400.3 ± 6.1	0.9996	3.4	11
AO 2246	0.02–2.0	56.91 ± 0.58	−292.8 ± 5.7	0.9998	2.7	9
AO 425	0.02–2.0	60.59 ± 0.71	503.3 ± 9.0	0.9997	2.4	8
AO 1790	0.02–2.0	38.05 ± 0.43	−180.9 ± 5.6	0.9998	3.6	12

* SE represents standard errors.

**Table 3:** Assessment of method precision and accuracy using spiking recovery experiments (n = 5).

Antioxidant	Spiked Level (ng/mL)	Simulant A Ultrapure Water			Simulant B 3% Acetic Acid Aqueous Solution			Simulant C 10% Ethanol Aqueous Solution		
		Recovery (%)	Intra-Day RSD (%)	Inter-Day RSD (%)	Recovery (%)	Intra-Day RSD (%)	Inter-Day RSD (%)	Recovery (%)	Intra-Day RSD (%)	Inter-Day RSD (%)
BHA	20	90.5 ± 5.3	5.8	6.3	94.8 ± 6.8	7.2	2.7	100.7 ± 5.3	5.3	3.4
	100	95.0 ± 2.2	2.3	2.7	96.7 ± 2.9	3.0	7.5	95.8 ± 6.5	6.8	4.7
	500	91.8 ± 4.0	4.4	7.4	97.1 ± 2.7	2.8	2.1	98.4 ± 2.1	2.1	3.5
AO 33	20	90.8 ± 3.7	4.1	1.3	96.1 ± 4.7	4.9	6.8	97.1 ± 5.7	5.9	4.9
	100	92.9 ± 2.9	3.1	6.4	90.7 ± 3.1	3.4	3.5	94.9 ± 5.2	5.5	3.6
	500	89.4 ± 2.3	2.6	0.9	93.3 ± 4.8	5.1	4.8	97.2 ± 5.1	5.2	5.3
AO 246	20	90.0 ± 5.9	6.5	3.4	101.6 ± 8.1	8.0	6.2	93.8 ± 3.8	4.0	2.6
	100	90.6 ± 1.8	2.0	7.1	98.3 ± 1.0	1.0	5.6	93.1 ± 2.7	2.9	4.2
	500	92.4 ± 2.9	3.1	1.8	97.1 ± 2.0	2.1	3.5	96.3 ± 7.1	7.4	3.7
AO 2246	20	93.4 ± 5.3	5.7	6.2	95.7 ± 5.0	5.2	6.9	97.6 ± 3.0	3.1	7.4
	100	94.1 ± 4.6	4.9	7.9	92.4 ± 3.6	3.9	6.4	93.2 ± 4.0	4.3	6.1
	500	93.6 ± 3.2	3.4	3.6	96.8 ± 5.2	5.4	1.2	98.4 ± 3.1	3.1	5.6
AO 425	20	89.7 ± 5.2	5.8	4.2	92.7 ± 2.6	2.8	4.2	96.3 ± 3.3	3.4	3.9
	100	90.0 ± 2.2	2.4	6.4	91.8 ± 3.0	3.3	5.4	95.1 ± 2.3	2.4	2.8
	500	91.3 ± 3.5	3.8	4.1	93.2 ± 2.2	2.4	5.6	97.6 ± 7.6	7.8	8.2
AO 1790	20	89.4 ± 2.2	2.4	4.3	91.8 ± 5.4	5.9	6.2	94.2 ± 4.9	5.2	7.7
	100	95.3 ± 3.0	3.1	3.0	97.5 ± 4.1	4.2	2.1	95.0 ± 3.6	3.8	3.2
	500	91.0 ± 2.3	2.5	3.5	96.4 ± 2.6	2.7	4.2	95.9 ± 2.3	2.4	3.5

**Table 4:** Comparison between the proposed method and reported methods in the literature.

Method	Matrix	Analytes	Sorbent Dosage	Total Time Spent	Sensitivity (LOD)	Recovery	Reference
d-SPFE-SFC	Simulants	6	8 mg	18 min	2.4–3.6 ng/g	89.4–101.6%	This research
d-SPE-HPLC-DAD	Tomato paste, etc.	4	500 mg	20 min	0.25–0.50 ng/g	>80%	[13]
M-SPE-UPLC-MS/MS	Plastics	2	15 mg	25 min	0.023–3.105 ng/g	70.6–102.3%	[14]
QuEChERS-LC-MS/MS	Salmon silage	3	42.5 g	27 min	12–15 ng/g	97–101%	[21]
DES-HPLC-UV	Simulants	3	0.15 g	30 min	0.15–0.25 μg/L	/	[24]
Ultrasonic extraction-LC-MS/MS	Simulants	2	/	35 min	0.0006–0.0012 mg/kg	96.66–98.05%	[34]

## Data Availability

The original contributions presented in this study are included in the article/Supplementary Material. Further inquiries can be directed to the corresponding author(s).

## Supplementary Materials

The following supporting information can be downloaded at: https://www.mdpi.com/article/10.3390/foods14132301/s1, Table S1: gradient elution program used in this study; Table S2: different gradient elution programs; Table S3: the application of the proposed method in real samples (n = 3).
