# Supplementary material for: Ultrasound-Assisted Dispersive Solid-Phase Filter Extraction Coupled with Green Supercritical Fluid Chromatography Methodology for Simultaneous Determination of Hindered Phenolic Antioxidant Migration from Food Contact Materials
Source: Foods. 2025 Jun 28;14(13):2301. doi: 10.3390/foods14132301 (PMC12248536; doi:10.3390/foods14132301)
Supplement: Supplementary file 1 [file foods-14-02301-s001.zip › foods-3720577-supplementary.pdf]

foods

MDPI

Ultrasound-assisted dispersive solid-phase filter extraction coupled with green supercritical fluid chromatography methodology for simultaneous determination of hindered phenolic antioxidants migration from food contact materials

Shaojie Pan [1], Chaoyan Lou [1,*], Xiaolin Yu [1], Kaidi Zhang [1], Kai Zhang [2], Lei Jiang [3] and Yan Zhu [4]

1 College of Quality and Standardization, China Jiliang University, Hangzhou 310018, China
2 Ningbo Key Laboratory of Agricultural Germplasm Resources Mining and Environmental Regulation, College of Science and Technology, Ningbo University, Ningbo 315300, China
3 Zhejiang Institute of Quality Sciences, Hangzhou 310018, China
4 Department of Chemistry, Zhejiang University, Hangzhou 310028, China

Table S1. Gradient elution program used in this study.

Time (min)	Flow rate (mL/min)	Solvent A ($Sc\text{-}CO_2$, %)	Solvent B (CH_3OH, %)
initial	1.5	95	5
4.00	1.5	95	5
4.10	1.5	90	10
6.00	1.5	90	10
6.10	2.0	90	10
10.00	2.0	90	10

Table S2. Different gradient elution programs.

Time (min)	Gradient 1		Gradient 2		Gradient 3	
	Flow rate (mL/min)	Modifier (%)	Flow rate (mL/min)	Modifier (%)	Flow rate (mL/min)	Modifier (%)
Initial	1.5	5	1.5	5	1.5	5
4.00	1.5	5	1.5	5	1.5	5
4.10	1.5	10	1.5	10	1.5	5
6.00	1.5	10	1.5	10	1.5	5
6.10	1.5	10	2.0	10	2.0	5
End	1.5	10	2.0	10	2.0	5

Table S3. The application of the proposed method in real samples (n=3).

Sample number	Type of simulants	Antioxidant content (mg/kg)					
		BHA	AO 33	AO 246	AO 2246	AO 425	AO 1790
1	Simulant A	ND[a]	ND	ND	ND	ND	ND
	Simulant B	ND	ND	ND	ND	ND	ND
	Simulant C	ND	ND	ND	ND	ND	ND
2	Simulant A	ND	ND	1.062	1.039	ND	ND
	Simulant B	ND	ND	1.205	1.048	ND	ND
	Simulant C	ND	ND	1.397	1.183	ND	ND
3	Simulant A	ND	ND	ND	ND	ND	ND
	Simulant B	ND	ND	ND	ND	ND	ND
	Simulant C	ND	ND	ND	ND	ND	ND
4	Simulant A	ND	ND	ND	ND	ND	ND
	Simulant B	ND	ND	ND	ND	ND	ND
	Simulant C	ND	ND	ND	ND	ND	ND
5	Simulant A	ND	ND	0.731	0.471	ND	ND
	Simulant B	ND	ND	0.756	0.684	ND	ND
	Simulant C	ND	ND	0.928	0.731	ND	ND
6	Simulant A	ND	ND	ND	0.781	ND	ND
	Simulant B	ND	ND	ND	0.792	ND	ND
	Simulant C	ND	ND	ND	0.834	ND	ND

[a]: Not detected (＜LOQ).
